# Incorporation of DNA methylation into eQTL mapping in African Americans

**Published:** 2021

**Authors:** Anmol Singh, Yizhen Zhong, Layan Nahlawi, C. Sehwan Park, Tanima De, Cristina Alarcon, Minoli A. Perera

**Affiliations:** Department of Pharmacology, Northwestern University Feinberg School of Medicine, Chicago, Illinois, United States of America

**Keywords:** genome-wide methylation, eQTLs, African Americans, Hepatocytes

## Abstract

Epigenetics is a reversible molecular mechanism that plays a critical role in many developmental, adaptive, and disease processes. DNA methylation has been shown to regulate gene expression and the advent of high throughput technologies has made genome-wide DNA methylation analysis possible. We investigated the effect of DNA methylation on eQTL mapping (methylation-adjusted eQTLs), by incorporating DNA methylation as a SNP-based covariate in eQTL mapping in African American derived hepatocytes. We found that the addition of DNA methylation uncovered new eQTLs and eGenes. Previously discovered eQTLs were significantly altered by the addition of DNA methylation data suggesting that methylation may modulate the association of SNPs to gene expression. We found that methylation-adjusted eQTLs that were less significant compared to PC-adjusted eQTLs were enriched in lipoprotein measurements (FDR=0.0040), immune system disorders (FDR = 0.0042), and liver enzyme measurements (FDR=0.047), suggesting that DNA methylation modulates the genetic regulation of these phenotypes. Our methylation-adjusted eQTL analysis also uncovered novel SNP-gene pairs. For example, we found that the SNP, rs1332018, was associated to *GSTM3. GSTM3* expression has been linked to Hepatitis B which African Americans suffer from disproportionately. Our methylation-adjusted method adds new understanding to the genetic basis of complex diseases that disproportionally affect African Americans.

## 1. Introduction

DNA methylation plays an important role in the regulation of gene expression which in turn affects many complex diseases and traits.^1^ Integration of DNA methylation into expression Quantitative Trait Loci (eQTL) mapping, can be challenging as the addition of SNP-based covariates is computationally intensive and multi-omics datasets with matching samples are sparse.^2^ Moreover, matching datasets in minority populations are nearly absent from public databases. DNA methylation patterns, in particular, are complex, vary greatly from sample to sample^3^, and change with environmental exposures.^4^ Therefore, DNA methylation studies can be hard to generalize. The advent of high throughput and next generation sequencing technologies, however, has made it possible for DNA methylation to be analyzed genome-wide.^4^ Several investigators have previously integrated genome-wide sequencing data and DNA methylation to uncover SNPs that significantly associate to CpG methylation status, called methylation QTLs (meQTLs).^5, 6^ These studies have found that DNA methylation plays a significant role in the onset of diseases and phenotypes such as obsessive-compulsive disorder and drug response.^5, 6^ Most of these studies have been conducted in populations of European ancestry.

The African American population is widely underrepresented in genetic studies. In GWAS studies, only 19% of individuals are non-European and less than 5% are non-European and non-Asian.^7^ While other eQTL mapping studies have used African American samples, the number of individuals have been very small, thus making them underpowered to adequately account for population specific variation. Furthermore, these studies did not account for DNA methylation as a SNP-based covariate.^7^ In this study, we perform the first investigation of the effect of DNA methylation on eQTL mapping in African Americans and evaluate methylation-adjusted eQTL associations to complex diseases, phenotypic traits, and metabolic traits. These findings may explain the role DNA methylation plays in health disparities observed in African Americans.

## 2. Methods

### 2.1. Cohort

**Fig. 1.**
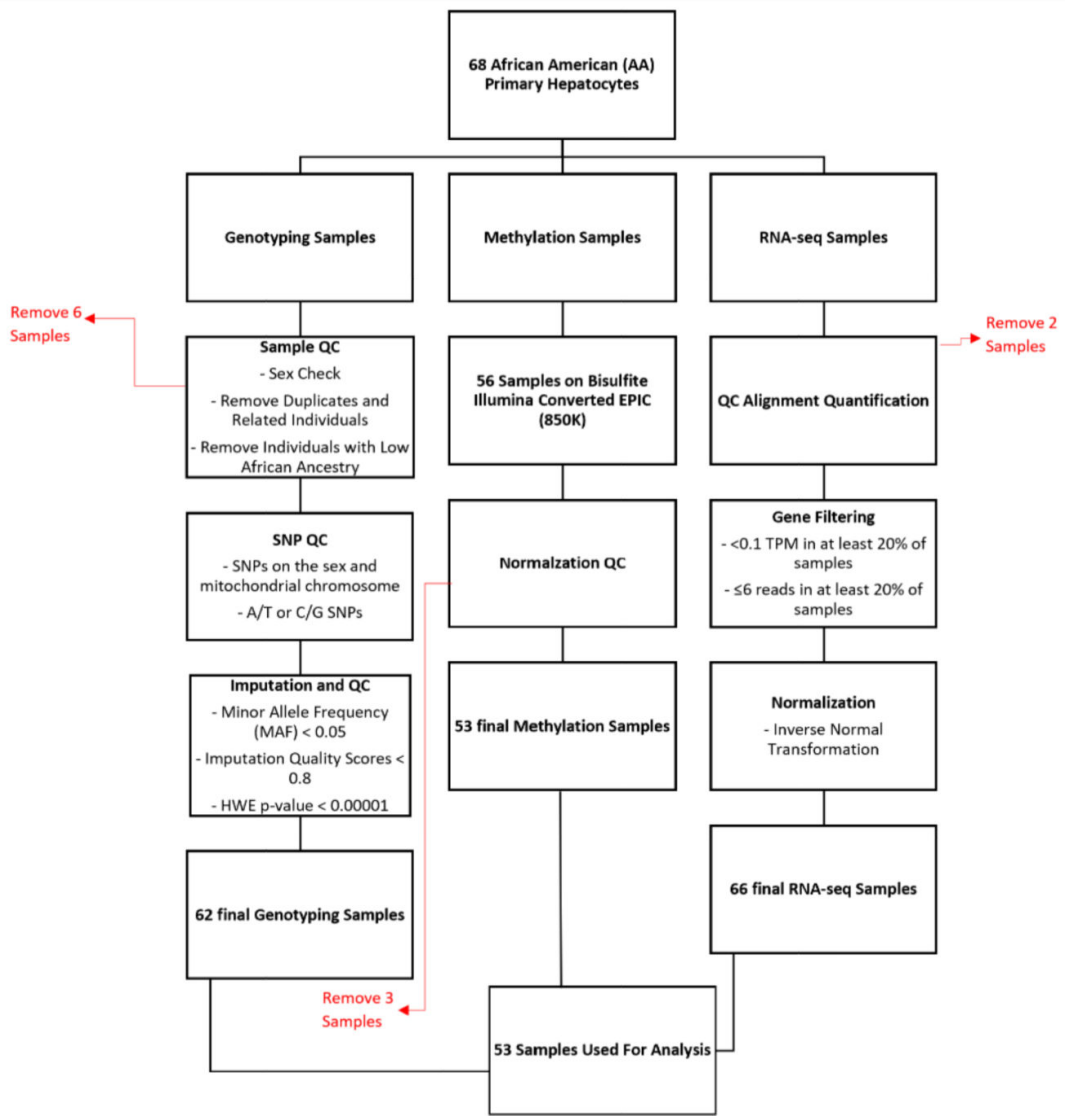
Flowchart showing the study design and the methods used in each dataset.

Sixty-eight African American hepatocyte cultures were acquired. After genotyping, DNA methylation quality control and RNA-sequencing quality control, 53 samples were used to conduct this analysis as shown in Fig. 1. Hepatocytes were either purchased from commercial companies (BioIVT, TRL, Life technologies, Corning, and Xenotech) or isolated from cadaveric livers using the same procedure described in Park et. al.^8^ All genomic, transcriptomic and methylome data were gathered from the same hepatocyte samples.

### 2.2. Genotyping, Imputation, and QC

DNA was extracted from each hepatocyte culture using Gentra Puregene Blood kit (Qiagen) and all the DNA samples were bar coded for genotyping. The SNPs were genotyped using the Illumina Multi-Ethnic Genotyping array (MEGA) at the University of Chicago Functional Genomics Core using standard protocols. The outputs were then created by Genome Studio using a 0.15 GenCall score as the cutoff. PLINK^9^ was then used to perform a sex check and to identify individuals with discordant sex information. The identity-by-descent method was used with a cut off score of 0.125 to identify duplicated or related individuals, where the cutoff score indicates third-degree relatedness. The following SNPs were excluded: SNPs on the sex and mitochondrial chromosome, A/T or C/G SNPs which may introduce flip-strand issues, SNPs with missing rate > 5% or failed Hardy-Weinberg equilibrium (HWE) tests (p < .00001), leaving 674,996 SNPs. Genotypes were phased using SHAPEIT and imputed with IMPUTE2. After imputations, SNPs were excluded for minor allele frequency < 0.05, imputation quality scores < 0.8, and HWE p-value < .00001, leaving 7,180,502 SNPs in the analysis.

### 2.3. RNA-sequencing and QC

Total RNA was extracted from each primary cell culture after three days of plating using the Qiagen RNeasy Plus mini-kit. Only the samples with RNA integrity number (RIN) score > 8 were sequenced. RNA-seq libraries were prepared using TruSeq RNA Sample Prep Kit, Set A (Illumina catalog # FC-122–1001) in accordance with the manufacturer’s instructions. Illumina HiSeq 2500 and HiSeq 4000 machines were used to prepare the cDNA libraries sequence and. This resulted in 50 million reads per sample (single-end 50bp reads).

Quality of the raw reads from FASTQ files was assessed by FastQC (v0.11.2). A per base sequence quality threshold of > 20 across all bases was set for the fastq files. STAR 2.5^10^ was used to align the reads to human Genome sequence GRCh38 and Comprehensive gene annotation (GENCODE version 25). Only uniquely mapped reads were retained and indexed by SAMTools 1.2.^11^ To assess the nucleotide composition bias, GC content distribution and coverage skewness of the mapped reads read_NVC.py, read_GC.py and geneBody_coverage.py from RNA-SeQC (2.6.4)^12^ were used. Lastly, Picard CollectRnaSeqMetrics was applied to evaluate the distribution of bases within transcripts. Fractions of nucleotides within specific genomic regions were measured and only samples with > 80% of bases aligned to exons and UTRs regions were retained for analysis.

### 2.4. Gene expression quantification

To quantify gene expression for the 17,992 genes used in the study a collapsed gene model was used, following the GTEx isoform collapsing procedure.^13^ The reads were mapped to genes referenced with Comprehensive gene annotation (GENCODE version 25) to evaluate gene-level expression using RNA-SeQC.^12^ The Bioconductor package, DESeq2 (version1.20.0)^14^ was used to supply HTSeq^15^ raw counts for the analysis of gene expression. DESeq2 was also used to perform principal component analysis (PCA). Using regularized log transformation, the counts were normalized. The two PC’s used in the study, PC1 and PC2, were plotted to visualize the expression patterns of the samples and two samples with distinct expression patterns were excluded as outliers.

The gene expression was normalized by the trimmed mean of M-values normalization method (TMM), which was implemented in edgeR.^16^ The TPM (transcript per million) was calculated by first normalizing the counts by gene length and then normalizing by read depth. The thresholds for gene expression values were set at < 0.1 TPM in at least 20% of samples and ≤ 6 reads in at least 20% of samples. Inverse normal transformation was used to normalize the expression values for each gene. The gene coordinates were remapped to hg19/GRCh 37 (GENCODE version 19) due to genotype imputation limitations.

### 2.5. Methylation Sample Preparation and QC

DNA was isolated from hepatocytes as described in Park et al.^8^ As shown in Fig. 1, 56 of the hepatocyte samples produced sufficient bisulfite-converted DNA for analysis. The Illumina MethylationEPIC BeadChip microarray (San Diego, Ca, USA), consisting of approximately 850,000 probes^17^ was used for methylation profiling from 56 AA hepatocytes that overlapped the samples used for gene expression analysis.

Methylation data QC and normalization was performed using the ChAMP R package (version 2.10.1)^18^ as previously described in Park et.al.^8^ This process removed: 9204 probes for any sample that did not have a detection p value <0.01, 1043 probes with a bead count <3 in at least 5% of samples, 49 probes that align to multiple locations as identified by Nordlund et al.^19^, 2975 probes with no CG start sites, and 17,235 probes located on X and Y chromosomes. After QC, three samples were excluded, resulting in 53 samples remaining in the analysis.

### 2.6. Methylation-adjusted eQTLs

The R package Matrix eQTL^20^ was used to determine the methylation site(s) that correspond to each SNP within a 2.5 kB window. CpG sites were then grouped together by SNP to determine the number of CpG sites on average at each SNP and to determine the pairwise correlation between CpG sites at each SNP. We used a weighted average based on the distance of the CpG site from the SNP to determine the methylation values for each SNP. If only one CpG site was linked to a SNP, then the weight of the CpG site would be:

(1) $$\text{w}=1-\left(\frac{d}{2500}\right)$$,

where d is the genomic distance (in base pairs) between the CpG site and the SNP and 2500 represents the 2.5kB window size used in this analysis. This weight would then be multiplied by the methylation value of the CpG site to get the normalized methylation value used in the analysis. This weighting system allowed proximal CpG sites to have a greater weight. If more than one CpG site was found within the 2.5kB region then each CpG site’s weight, *w*_i_, was calculated using equation (1) above and the final weight for each CpG site was calculated as:

(2) $$w_{f}=\frac{w_{i}}{\sum_{k=1}^{N}w_{k}}$$,

where N is the total number of CpG sites that correspond to a particular SNP and $\sum_{k=1}^{N}w_{k}$ represents the sum of the initial weights of all the CpG sites that correspond to that SNP. This calculation ensures the sum of the final weights of all CpG sites corresponding to a single SNP are equal to one. The SNP-based methylation value was then calculated by:

(3) $$\text{M}=\sum_{f=1}^{N}w_{f}*m_{f}$$,

where M is the SNP-based methylation value and $\sum_{f=1}^{N}w_{f}*m_{f}$ represents the weighted sum of all of the methylation values for the CpG sites corresponding to that SNP. These averaged methylation values used as a SNP based covariate and eQTLs were mapped using the LAMatrix R package.^21^ The methylation-adjusted eQTLs and PC-adjusted eQTLs were adjusted for sex, platform, batch, genotype-derived PCs 1 and 2, and 10 PEER variables estimated from normalized expression values as previously described in Zhong et.al.^7^ The genotype-gene expression associations within a *cis* region (1 Mb around the gene) were tested. PC-adjusted eQTLs (mapped in the same hepatocyte cultures) were compared to methylation-adjusted eQTLs to investigate if changes in the eQTL statistical significance or change in effect size (Spearman’s correlation).^21^ All relevant data are within the manuscript are available from the GEO (GSE124076 and GSE147628).

### 2.7. eQTL and GWAS overlap

To understand how the methylation-adjusted eQTLs may explain the underlying mechanisms in GWAS findings, the method presented in Zhong et. al.^7^ was used with some modifications. This included downloading the NHGRI/EBI GWAS Catalog file (v.1.0.2, 2019–03-22) and keeping only the associations that passed the genome-wide significant level (p<5e-8). Furthermore, the rsids were remapped from Build38 to Build37 using Ensembl API. The 1000 Genomes YRI population were used to extract all the variants in LD with the independent GWAS variants (r^2^ > 0.8) and the traits of the corresponding GWAS hits were put into 17 groups which corresponded to ontology-based trait categories.^22^ A false discovery rate (FDR) threshold of 0.05 was set as significant enrichment for an ontology. The methylation-adjusted eQTLs were split into three groups for this analysis: (i) eQTLs that were significant with PC-adjustment and increased in significance with methylation-adjustment, (ii) eQTLs that were not significant with PC-adjustment and became significant with methylation- adjustment (FDR<0.05), and (iii) eQTLs that were significant with PC-adjustment and became less significant with methylation-adjustment. These three groups of eQTLs were compared to the GWAS variants.

## 3. Results

Fifty-three African American hepatocyte samples were used in this analysis, with 28 (52.8%) males and 25 (47.2%) females. The age (mean ± standard deviation) of the cohort was 39 ± 18.4 years old. To account for methylation in this eQTL mapping analysis, LAMatrix was used.^21^ Instead of incorporating local ancestry into the analysis as previously done^7^, DNA methylation was used in its place. LAMatrix was chosen because the R package has increased power and controls false positives when gene expression differs by locus-specific covariate, such as methylation.^21^

### 3.1 Methylation-adjusted *eQTLs vs PC-adjusted eQTLs*

**Fig. 2.**
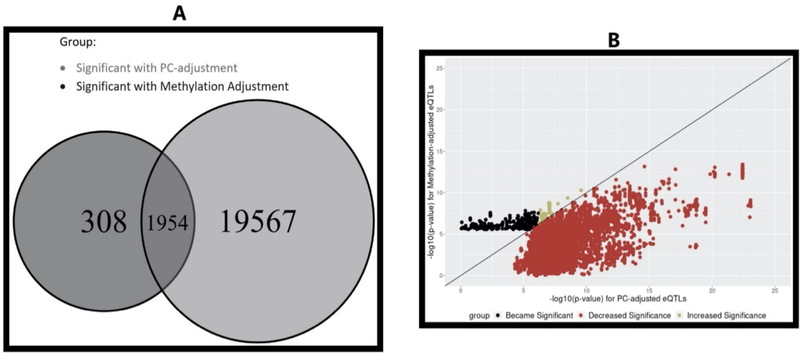
Methylation-adjusted eQTLs as compared to PC-adjusted eQTLs A) The Venn-Diagram showing the number of eQTLs that are significant with methylation-adjustment, significant with PC-adjustment, and significant in both analyses. B) Comparison of the p-values of the 308 eQTLs that became significant with methylation-adjustment (black), 11,485 that were significant with PC-adjustment and decreased in significance with methylation-adjustment (red), and 50 that were significant with PC-adjustment and increased in significance with methylation-adjustment (gold).

Out of the 7,180,502 total SNPs in the dataset, 2,494,181 SNPs had at least one CpG site within the 2.5 kB window, with an average of 3.08 CpG sites per SNP (ranging between of 1 to 95 CpG sites per SNP). We identified 2,296 eQTLs with methylation-adjustment at an FDR threshold of 0.05. To ascertain if any methylation-adjusted eQTLs resulted in the novel discovery of regulatory variation, we compared significant methylation-adjusted eQTLs (FDR<0.05) against significant PC-adjusted eQTLs (FDR<0.05). This comparison resulted in 308 unique methylation-adjusted eQTLs that were not found with PC-adjusted analysis, and 1,954 eQTLs which were common to both analyses. The remaining 19,567 found in PC-adjustment were not significant in this analysis (Fig. 2A). The comparison revealed that there were 11,485 eQTLs that were significant with PC-adjustment and decreased in significance with methylation-adjustment and 50 eQTLs that were significant with PC-adjustment increased in significance with methylation-adjustment (Fig. 2B). We compared the effect size for methylation-adjusted eQTLs (all methylation-adjusted eQTLs and by the groups defined in Fig. 2B) versus PC-adjusted eQTLs. All groups showed high correlation of effect size (Spearman’s correlation = 0.32–0.42, p < 2.2e-6, data not shown).

### 3.2 GWAS Associations for Methylation-adjusted eQTLs

We overlapped the methylation-adjusted eQTLs with SNPs in previously reported GWAS. Variants, from NHGRI-EBI GWAS catalog, or their tagging variants (r^2^ > 0.8, 1000 Genomes YRI population) were used to determine the overlap with h the methylation-adjusted eQTLs. To analyze the effect of methylation even further, the methylation-adjusted eQTLs were broken into three groups: (i) eQTLs that are only significant with methylation-adjustment, (ii) eQTLs that were significant with PC-adjustment but became more significant with methylation-adjustment, and (iii) eQTLs that were significant with PC-adjustment but became less significant with methylation-adjustment. In total, there were 285 GWAS associations that intersect with methylation-adjusted eQTLs across the three groups.

#### 3.2.1. Group 1: eQTLs that were only significant with methylation-adjustment (N = 308)

**Fig. 3.**
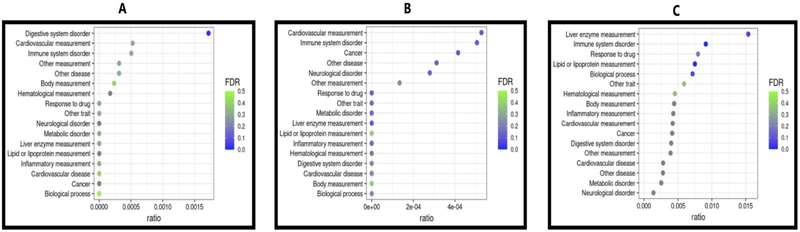
Enrichment of methylation-adjusted eQTLs in GWAS findings A) eQTLs, that were only significant with methylation-adjustment. B) eQTLs, that were significant with PC-adjustment and increased in significance with methylation-adjustment. C) eQTLs, that were significant with PC-adjustment and decreased in significance with methylation-adjustment. The X-axis represents the proportion of SNPs within each category that were within each group and FDR for enrichment is shown by the color of the dot.

For eQTLs that were only significant with methylation-adjustment, 16 GWAS associations were found that intersected with these eQTLs. There was significant enrichment for digestive system disorders (FDR = 0.011), as shown in Fig. 3A. One of the eQTLs enriched for digestive system disorders, rs11546996, was associated with primary biliary cirrhosis.^23^ Due to the intergenic location of rs11546996, the causal gene was reported as *SPIB* in this study. However, our analysis associated rs11546996 to *PNKP* (P-value = 1.05e-6, FDR = 0.026) thereby potentially identifying a new causal gene for primary biliary cirrhosis by accounting for methylation in eQTL mapping.

#### 3.2.2. Group 2: eQTLs that were significant with PC-adjustment and increased in significance with methylation-adjustment (N = 50)

For eQTLs that were significant with PC-adjustment and increased in significance with methylation-adjustment, eight GWAS associations intersected with this group of eQTLs. No significantly enriched was found (Fig. 3B). This may be due to the very small number of eQTLs in this group.

#### 3.2.3. Group 3: eQTLs that were significant with PC-adjustment and decreased in significance with methylation-adjustment (N = 11,485)

For eQTLs that were significant with PC-adjustment and decreased in significance with methylation-adjustment, 261 GWAS associations intersected with eQTLs in this group. There was significant enrichment for lipid or lipoprotein measurements (FDR = 0.0040), immune system disorders (FDR = 0.0042), and liver enzyme measurements (FDR = 0.047) (Fig. 3C). This suggests that these SNPs may be associated to susceptibility of disease, but that susceptibility may be modulated by DNA methylation.

Novel SNP-gene associations were also found. Two examples are rs7528419 and rs12740374, which are associated with *SORT1,* a gene known to influence LDL-cholesterol levels and lipid/lipoprotein measurements.^24^ When accounting for DNA methylation the p-value of these two SNP-gene pairs increased from 1.99e-9 for both to 4.92e-8 and 1.25e-7, respectively. The FDR also increased from 3.01e-5 for both to 3.12e-3 and 6.08e-3, respectively. Furthermore, both SNPs had proportion of DNA methylation ranging from 0.12 to 0.33. Although these SNP-gene pairs remained significant with methylation-adjustment, their significance decreased dramatically indicating that methylation, near these SNPs, may play a role in the association between these SNPs to lipid phenotypes. This suggests that DNA methylation should be considered when assessing genomic risk of LDL-cholesterol levels and cholesterol-related diseases, such as myocardial infarction.

The methylation-adjusted eQTL, rs9296736 associated with the expression of *MLIP,* was previously found to be associated with liver enzyme measurements.^25^ High levels of liver-enzymes in plasma are widely associated with an increased risk for developing many diseases including cirrhosis and cardiovascular disease.^25^ This SNP-gene pair decreased in significance considerably when it was adjusted for methylation. The p-value and FDR of this methylation-adjusted eQTL went from 2.08e-9 and 3.13e-5 with PC-adjustment to 0.037 and 0.94, respectively, with methylation-adjustment. For this SNP-gene pair, rs9296736 was highly methylated, with proportion of DNA methylation ranging from 0.89 to 0.97. This result suggests that the association of rs9296736 to *MLIP* and liver enzyme measurements may depend on the DNA methylation landscape.

### 3.3. Discovery of eGenes associated to disease traits using methylation-adjusted eQTL mapping

**Fig. 4.**
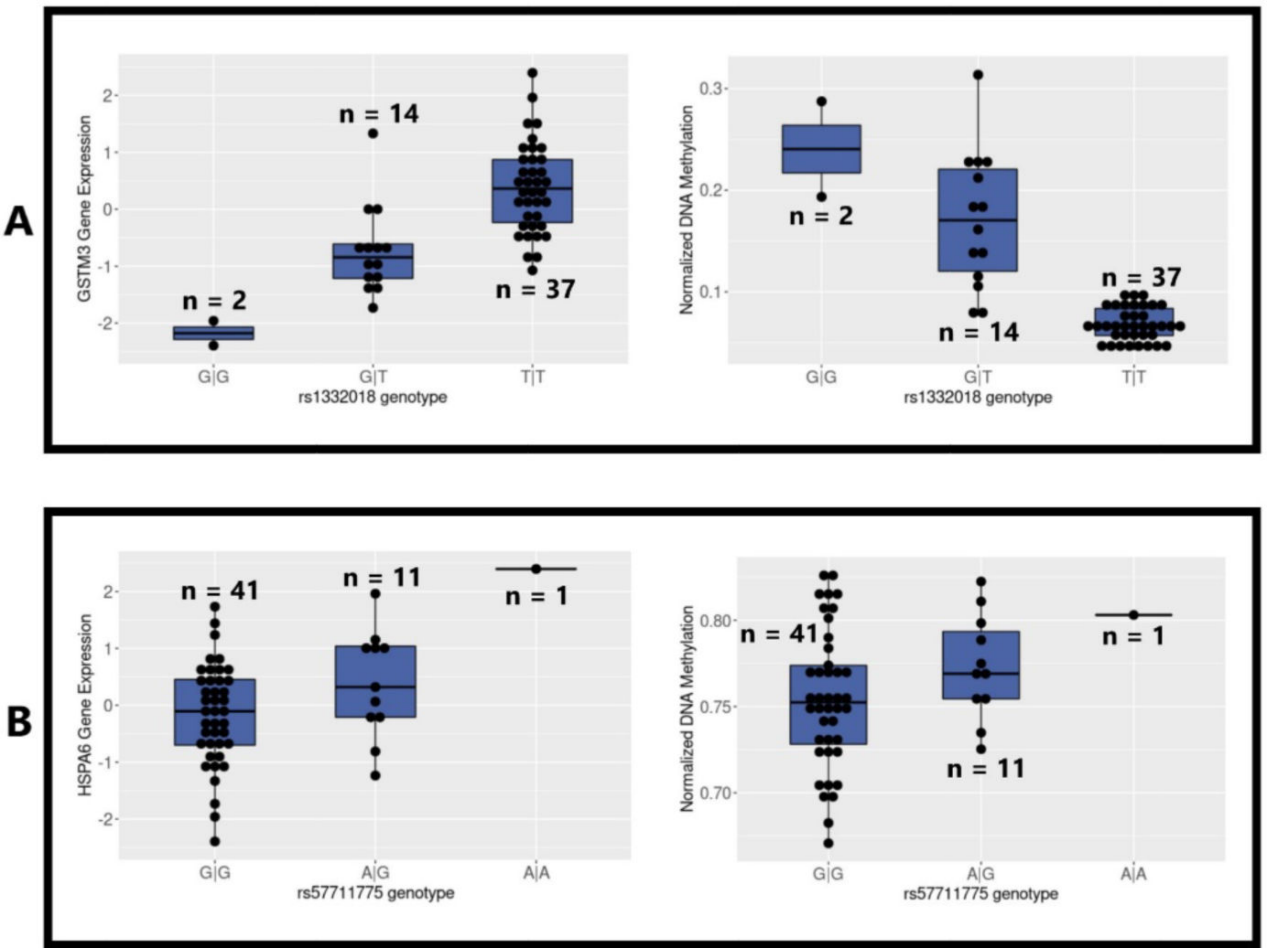
Boxplots of genotype vs gene expression and DNA methylation for *GSTM3 and HSPA6*. A) A significant increase in *GSTM3* gene expression (p = 1.1e-6) and a significant decrease in DNA methylation (p = 9.2e-13) are associated with rs1332018. The number of individuals (n) is shown for each genotype. B) A significant increase in *HSPA6* gene expression (p=0.0099) and an increase in DNA methylation (p=0.20) are associated with rs57711775. The number of individuals (n) is shown for each genotype.

There were 179 eGenes found through methylation-adjusted eQTL mapping (FDR<0.05) of which 80 eGenes that were not significant with PC-adjustment. Two of these eGenes, *GSTM3* (FDR = 0.014) and *HSPA6* (FDR = 0.029), have been associated to disease traits such as Hepatitis B (HBV) for *GSTM3* and Hepatocellular Carcinoma (HCC) for both eGenes.^26–29^ African Americans have a higher incidence and worse outcomes of HBV and HCC when compared to other demographics.^30, 31^ Since these eGenes were not significant with PC-adjusted eQTL mapping, they may explain how methylation plays a role in the health disparities observed in African Americans. As shown in Fig. 4A, there is a significant assocication between rs1332018 genotype and *GSTM3* expression as well as rs1332018 genotype to DNA methylation. From this we can see that the T allele is associated with both increased gene expression and lower DNA methylation. A total of 18 CpG sites contributed to this association. As shown in Fig. 4B, there is also relationship between *HSPA6* gene expression and DNA methylation with the A allele associated with both increased gene expression and increased DNA methylation, though the later did not reach statistical significance. A total of 7 CpG sites contributed to this association.

## 4. Discussion

Through the integration of DNA methylation into eQTL mapping, we showed how methylation potentially plays a critical role in SNP-gene associations as well as the association of these eQTLs to diseases and metabolic traits. Our analysis was aided by using the computationally efficient R package, LAMatrix, which allows for the addition of a SNP based covariate to eQTL mapping. Additionally, our use of data from African Americans aided in the discovery of new regulatory variants as this population is more genetically diverse than European ancestry populations. Previous meQTLs studies have shown that SNPs can affect the methylation status of nearby CpGs, not only CpGs that overlap the SNP location. Shultz et. al. showed that SNPs within 0.2Mb of a CpG can significantly associate with methylation status.^32^ We used a weighted approach which assumes that SNPs have a larger effect on closer CpG sites as previous studies showed a decrease in the association p-values of meQTLs with distance.^33, 34^ In our analysis we accounted for methylation within a 2.5Kb window. Larger window sizes may be more appropriate, but as no previous study has incorporate methylation into eQTL mapping we took a conservative approach.

We found unique eGenes in our analysis that were not found by eQTL mapping with only PC-adjustment. Two of these eGenes, *GSTM3* and *HSPA6*, are associated with diseases such as HBV for *GSTM3* and HCC for both eGenes.^26–29^ These are diseases that disproportionately affect African Americans.^30, 31^ *GSTM3* has also been associated to oxidative stress and specifically several studies have found that epigenetic suppression of *GSTM3* in HBV-infected cells causes oxidative stress^27, 28^, which can lead to HCC.^26^ Furthermore, other studies showed that *GSTM3* expression was lowered with promoter hypermethylation^35^ and in chemical-induced HCC.^36^ This finding agrees with the previous studies mentioned, showing that epigenetic suppression of *GSTM3* leads to HCC in HBV-infected cells.^26–28^ We found a significant inverse relationship between *GSTM3* expression and DNA methylation around rs1332018. This suggests that individuals with rs1332018 genotypes that have a lower *GSTM3* expression and higher methylation may be at a higher risk for HCC. *HSPA6* was also found to be overexpressed in human HCC tissues and a potential risk factor for HCC reccurence.^29^ We found that the expression of *HSPA6* increased with methylation around rs57711775, which could mean that methylation potentially plays a role in upregulating *HSPA6*. Furthermore, the A allele of rs57711775 that is associated with higher *HSPA6* expression and higher methylation in our analysis. This SNP is not found in European ancestry populations according to the Ensembl database. Thus, we potentially elucidated a causal variant and risk allele for HCC specific to African Americans by incorporating methylation into this analysis. As has previously been reported, the direction of effect of DNA methylation is dependent on the location of methylation.^37, 38^ Previous studies have shown that methylation within the transcriptional start site of the promoter is well known to repress gene expression while methylation within the gene body results in more variable expression.^37, 38^ Therefore, both *GSTM3* expression and *HSPA6* expression may contribute to the onset of HCC in African Americans.

In our GWAS enrichment, we found a significant enrichment for digestive system disorders for the eQTLs significant only with methylation-adjustment and a significant enrichment for lipid or lipoprotein measurements, immune system disorders, and liver enzyme measurements for the eQTLs that are less significant with methylation-adjustment. Both immune-related phenotypes and lipid and lipoprotein measures differ by population and may contribute to disease disparities. Our findings suggest that methylation may play a role in these diseases. Further studies are needed to determine if DNA methylation around these specific SNPs and genes differ between populations. This analysis also revealed an interesting association with eQTLs that were only significant after methylation-adjustment. The SNP, rs11546996, a SNP associated with primary biliary cirrhosis, was a methylation-adjusted eQTL for *PNKP*. In a previous GWAS study, a causal SNP-gene association for primary biliary cirrhosis was found with rs11546996 and the causal gene was assumed to be *SPIB,* as it is the closest gene.^23^ Since our study specifically looked at gene expression in hepatocytes, a tissue relevant for this disease, we may have found a potentially novel SNP-gene pair associated with primary biliary cirrhosis whose expression is regulated by both DNA methylation and gene variation. *PNKP* has also been associated with repairing DNA after damage from oxidative stress^39^, so rs11546996 could be a SNP that effects this process.

There were several limitations to our study. First, we were only able to include 53 samples into this analysis and hence our analysis was underpowered. Second, we assessed DNA methylation with the Illumina EPIC array which is limited to the CpG sites chosen for the chip. Unmeasured DNA methylation may have effects on eQTLs that were not captured by our analysis. Third, our results, compared to the findings in the entire GWAS catalog, are only applicable to diseases in which hepatocytes play a key role. Our findings may not be generalizable to other cell or tissue types. Finally, we have assumed that DNA methylation closer to the SNP is more likely to influence eQTL mapping, however this may not always be the case. Additionally, we do not account for differences in effect size in our method. With greater meQTL analysis in relevant cell types and populations, we may be able to weight SNP/DNA methylation interactions more precisely as a SNP-based covariate.

In conclusion, this is the first study to explore the effect of DNA methylation in eQTL mapping in African Americans. The African American demographic is widely underrepresented in genetic studies and their greater genetic diversity may allow us to find novel SNP-gene pairs as well as population specific SNPs. Our findings can be used to understand how DNA methylation potentially plays a role in complex diseases, phenotypic traits, and metabolic traits in African Americans.
